# Studies on the Occurrence of Viruses in Planting Material of Grapevines in Southwestern Germany

**DOI:** 10.3390/v13020248

**Published:** 2021-02-05

**Authors:** Noemi Messmer, Patricia Bohnert, Stefan Schumacher, René Fuchs

**Affiliations:** State Institute of Viticulture and Enology, Merzhauser Straße 119, 79100 Freiburg, Germany; Noemi.Messmer@wbi.bwl.de (N.M.); Patricia.Bohnert@wbi.bwl.de (P.B.); Stefan.Schumacher@wbi.bwl.de (S.S.)

**Keywords:** grapevine, Grapevine leafroll-associated virus 1 (GLRaV-1), Grapevine leafroll-associated virus 3 (GLRaV-3), Grapevine fanleaf virus (GFLV), Arabis mosaic virus (ArMV), Grapevine fleck virus (GFkV), Grapevine Pinot gris virus (GPGV)

## Abstract

Viral diseases in viticulture lead to annual losses in the quantity and quality of grape production. Since no direct control measures are available in practice, preventive measures are taken to keep the vines healthy. These include, for example, the testing of propagation material for viruses such as *Arabis mosaic virus* (ArMV), *Grapevine fanleaf virus* (GFLV) or *Grapevine leafroll-associated virus* 1 (GLRaV-1) and 3 (GLRaV-3). As long-term investigations have shown, GLRaV-1 (2.1%) occurs most frequently in southwestern German wine-growing regions, whereas GLRaV-3 (<0.1%) is almost never found. However, tests conducted over 12 years indicate that there is no general decline in virus-infected planting material. Thus, it can be assumed that a spread of the viruses via corresponding vectors still takes place unhindered. Beyond the examinations regulated within the German Wine Growing Ordinance, one-time tests were carried out on *Grapevine Pinot gris virus* (GPGV). This analysis showed that GPGV was found in 17.2% of the samples.

## 1. Introduction

Grapevine is the perennial crop with the highest number of intracellular pathogens, of which 86 are viruses [1,2]. Viral infections are particularly insidious because they may be latent and direct plant protection measures are not available. For this reason, preventive measures must be used first and foremost to avoid virus infections. These include the use of healthy planting material, containment of viral vectors and breeding of resistant or tolerant varieties [3].

Using healthy starting material is a crucial step in practicing integrated plant management in viticulture. The sanitary status of planting material is particularly essential because grapevine is propagated vegetatively. From 1986, that measure has been regulated in Germany by the Wine Growing Ordinance (Rebenpflanzgutverordnung (RebPflV)) [4]. The frequency and quantity of virus testing of propagating material depends on the category for which vine nurseries are registered. However, for all categories, the regulation stipulates that the planting material must be tested for the following viruses:

- *Arabis mosaic virus* (ArMV)
- *Grapevine fanleaf virus* (GFLV)
- *Grapevine leafroll-associated virus* 1 (GLRaV-1)
- *Grapevine leafroll-associated virus* 3 (GLRaV-3)
- *Grapevine fleck virus* (GFkV) (rootstock only)

ArMV and GFLV both belong to the genus *Nepovirus* and are the most typical representatives of this genus in European vineyards [5]. Virus transmission occurs through two types of nematodes, namely *Xiphinema index* and *Xiphinema diversicaudatum* [6,7]. GLRaV-1 and -3 both belong to the genus *Ampelovirus*. More than ten other viruses are also associated with causing leafroll in grapevine [8]. Viral vectors are multiple mealybugs and soft scale insects, widely spread in Europe [9]. GFkV is assigned to the genus *Maculavirus*. No vectors have been found for this virus to date. However, Martelli and Boudon-Padieu [9] listed several references reporting natural field spread in symptomatic plants. While ArMV, GFLV, GLRaV-1, and GLRaV-3 cause characteristic symptoms in grapevine, GFkV affects plants only in the case of a co-infection with at least one of the other viruses [10].

All listed viruses have in common that they are graft transmissible and can cause severe biological, and thus economic, losses [11,12,13]. The regulation aims to trade only pathogen-free and healthy planting material by the negative selection of infected material. This measure is the cornerstone for a sustainable and successful cultivation strategy as it prevents an exponential entry of infected vines. The propagation material screening is also intended to prevent vector insects from getting in contact with their corresponding virus. Most insects are not in themselves a threat to grapevine as a regular occurrence [14]. However, in combination with an incorporated virus, their risk potential is significantly increased.

From 1986 onwards, the regulation has been amended several times to meet new requirements and current circumstances. Since 2009, the State Institute of Viticulture and Enology (WBI) in Freiburg, Germany, has had the task of carrying out the official virus testing in southwest Germany. The WBI diagnostic laboratory receives wood samples from registered propagation vineyards from the regions concerned. These are tested for viruses utilizing enzyme-linked immunosorbent assay (ELISA).

This study summarizes twelve years of virus testing at the WBI of the pre-basic, basic, and certified propagating material category, concentrating on scion material. The results showed that:

1. GLRaV-1 was the most abundant virus in Baden-Wuerttemberg followed by GFLV and ArMV. GLRaV-3 was rarely detected.
2. GLRaV-1 infections were more widespread in certified plant material than in pre-basic and basic material.
3. Wuerttemberg showed higher GLRaV-1 and GFLV incidences than Baden. Significant differences exist between pre-basic and basic planting material, but not between certified material.
4. Virus infections are more frequent in scion than in rootstock planting material.

## 2. Materials and Methods

### 2.1. Definition of Propagating Material Categories

According to the German Wine Growing Ordinance [4], grapevine propagation materials can be assigned to one of the following four categories upon request to the responsible authority:

1. Pre-basic
2. Basic
3. Certified
4. Standard

Depending on the category, the phytosanitary requirements for planting material differ significantly. While vines of all categories must be tested for the absence of ArMV, GFLV, GLRaV-1, and GLRaV-3 (GFkV must be monitored only in rootstocks), pre-basic vines are subject to the strictest requirements regarding sampling quantities and retesting intervals. The strictness of the guidelines decreases in the above order. This study will not consider plant material of the standard category, which has only been marketed for commercial purposes in Germany since 2017 following the amendment of the Wine Growing Ordinance [4].

#### 2.1.1. Pre-Basic Planting Category

Pre-basic plant material must be derived from a vineyard with plants of a generation prior to the basic plant material of the variety or clone indicated. Planting material must be obtained by the breeder or under his/her supervision and according to his/her instructions in accordance with the principles of conservation breeding. Samples for virus testing are taken from each plant. Five plants are pooled into one mixed sample. Vineyards must be retested in five-year intervals. Virus positive plants must be removed.

#### 2.1.2. Basic Planting Category

Basic plant material must be derived from a vineyard planted with approved pre-basic plant material of the variety or clone indicated. Planting material must be obtained by the breeder or under his/her supervision and according to his/her instructions in accordance with the principles of conservation breeding. Samples are taken from each plant. Ten plants are pooled together into one mixed sample for virus testing. Vineyards are first sampled three years after planting and must be retested at least once every six years. Virus-positive plants must be removed.

#### 2.1.3. Certified Planting Category

Certified plant material must be derived from a vineyard planted with basic or approved pre-basic plant material of the variety or clone indicated. Samples are taken from every twentieth plant. Ten plants are pooled together into one mixed sample for virus testing. Vineyards are first sampled five years after planting and must be retested at least once every ten years. Virus-positive plants must be removed and must not exceed five out of one hundred plants.

### 2.2. Plant Material for Virus Testing

The vineyards sampled during this study were selected by the breeding departments of the State Institute of Viticulture and Enology (WBI, Freiburg, Germany) for the Baden region and the State Education and Research Institute for Viticulture and Pomology (LVWO, Weinsberg, Germany) for the Wuerttemberg region. The sampled vine nurseries differ in planting time, grape variety, size, category of propagation material, and geographical position. Wood samples were collected from vineyards by officially trained personnel. Therefore, one-year-old shoots from a stem-near position were cut off and shortened to approximately 15 cm in length and stored at 4 °C until further processing. At the laboratory, thin slices were cut from the lower part of the shoots using a custom-designed machine (Wagner Hydraulik und Antrieb GmbH, Ehrenkirchen, Germany). Wood from one bundle was pooled into one sample for virus testing and tested immediately as described in Section 2.3. Depending on the propagating category, shoots were collected in bundles of five (pre-basic) or ten (basic, certified) either from each plant (pre-basic, basic) or only from every twentieth grapevine (certified), as mentioned above. Approximately 20% of the vineyards in this study were tested at least twice between 2009 and 2020.

### 2.3. Virus Detection

Samples were tested by double-antibody sandwich enzyme-linked immunosorbent assay (DAS-ELISA). Assays were performed with the commercially available equipment and products of BioReba (Reinach, Switzerland) following the protocols provided by the manufacturer. One gram of pooled wood samples was arranged in extraction bags and homogenized in 1:10 (*w*/*v*) customized “Grapevine” extraction buffer using the homogenizer HOMEX (BioReba, Reinach, Switzerland). Scion samples were tested for ArMV, GFLV, GLRaV-1, and GLRaV-3 following the manufacturer’s recommendations. Samples from rootstock plants were also tested for GFkV. In 2018, scion and rootstock samples were additionally tested for GPGV. Positive and negative controls were obtained from the WBI virus collection located in Freiburg, Germany. ELISA plates were evaluated photometrically after 30 and 60 min, using an Infinite F50 reader and Magellan™ software (Tecan Trading AG, Maennedorf, Switzerland). Samples were considered positive if the absorbance value was twice the value of the negative control sample.

### 2.4. Statistical Analysis

Data were analyzed using the statistical software R (Version 1.2.5001, Boston, MA, USA). Chi-square tests were conducted to analyze hypotheses that relied only on nominal data. An alpha level of 0.05 was chosen for all statistical tests.

## 3. Results

Since 2009, the official virus testing for the wine-growing areas Baden and Wuerttemberg has been executed at the WBI following the legislative requirements of the German Wine Growing Ordinance [4]. Until 2020, a total amount of 512,705 grapevine plants were analyzed with the aim of approving only virus-free propagation material for the market. Of the samples, 469,199 were assigned to scion material and 43,506 to rootstock material, resulting in 18,756 and 4906 pooled samples, respectively. The following results refer to scion propagating material unless indicated differently. All shown values refer to the number of pooled samples. Mixed infections were not considered, as only 4% of the tested vineyards were affected.

### 3.1. Scion Plant Material 2009–2020

**Figure 1:**
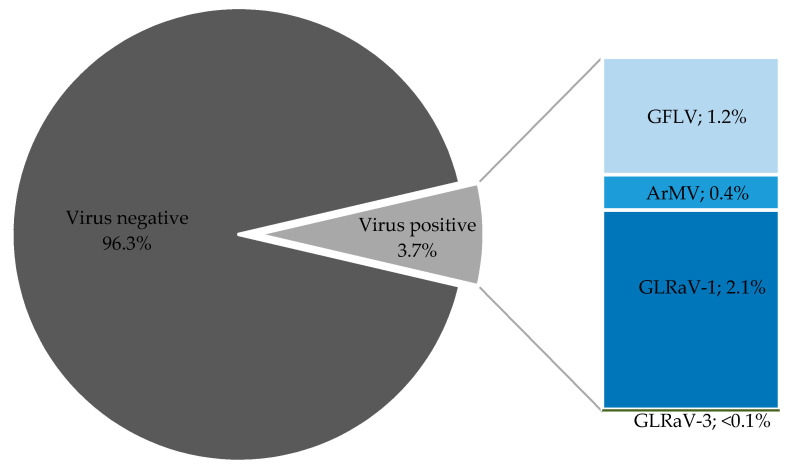
Only 3.7% of samples were found to be virus infected in 2009–2020. The left part of the chart shows the overall amounts of virus-negative (dark gray color) and virus-positive (light gray color) scion samples. The right chart represents the proportions of viruses found inside the 3.7% of virus-infected scion samples. Samples were tested for *Grapevine fanleaf virus* (GFLV); *Arabis moasic virus* (ArMV); *Grapevine leafroll asscoiated virus 1* (GLRaV-1); *Grapevine leafroll associated virus 3* (GLRaV-3). Mixed infections were not considered.

Over the last 12 years, 96.3% of the tested samples were free from ArMV, GFLV, GLRaV-1, and GLRaV-3 (Figure 1). Consequently, 3.7% of the samples were found to be virus infected. The proportions of the verified viruses vary significantly, *X*^2^(3, N = 18756) = 511.90, *p* < 0.01. GLRaV-1, the most abundant virus, was detected in 2.1% of the tested samples. GFLV was found in 1.2% and ArMV in 0.4% of the samples. GLRaV-3 was identified in less than 0.1% of the samples and thus was the rarest virus found.

### 3.2. Virus Occurrence in Propagating Categories and Over Time

Further details of the testing can be extracted if the data are separated by category of propagating material (Table 1). In pre-basic plant material, GLRaV-1 (1.2%), GFLV (0.9%), and ArMV (0.3%) differed only marginally, while GLRaV-3 was found in only one sample. The high incidence of GLRaV-1 was due to one vineyard with 64 positive samples. In basic propagating material, GFLV was found in 1.7% of the samples, GLRaV-1 was present in 1.4%, and ArMV in eight samples (0.1%). GLRaV-3 was not detected in any sample. Interestingly, the GLRaV-1 proportion was highest in certified plant material, where 8.0% of the samples tested positive for this virus, *X*^2^ (2, N = 18756) = 430.09. *p* < 0.001. GFLV and ArMV were both found in 1.3% of the samples. Three pooled samples showed a positive signal for GLRaV-3, which refers to 0.1% of samples (Table 1).

Looking at the individual years, the occurrence of the different viruses varies considerably. GLRaV-1, GFLV, and ArMV seem to increase in specific years and then not reappear for several years. This becomes particularly visible within certified plant material.

Since the grapevine cultivar Lemberger is a frequent carrier of GLRaV-1 [15], the virus distribution inside the three categories was again considered separately for Baden and Wuerttemberg (Table 2). While ArMV (0.3%) and GLRaV-3 (0.0%) were distributed similarly in Baden and Wuerttemberg, GFLV (0.9% vs. 2.3%) and GLRaV-1 (1.5% vs. 3.8%) were identified more frequently in Wuerttemberg, *X*^2^ (1, N = 19232) = 165.23. *p* < 0.001. These differences are caused by higher virus proportions in pre-basic and basic plant material from Wuerttemberg, *X*^2^ (1, N = 9680) = 56.14, *p* < 0.01; *X*^2^ (1, N = 6259) = 74.652, *p* < 0.001. Virus contents in certified material are similar in both regions, *X*^2^(1, N = 3293) = 0.56, *p* = 0.45).

### 3.3. Multiply Tested Vineyards

Due to the regulations for virus testing within the German Wine Growing Ordinance [4], vineyards with a certain number of virus-positive tested plants are often dismissed for propagation purposes. Therefore, of the total of 1089 plots, only 100 were tested twice and 10 three times. However, these vineyards are valuable to find out if there are viral inputs from outside. In 10 (9%) of the vineyards retested after 5 or 6 years, GLRaV-1 infections were detected in one of the repeat tests, indicating a later infection event by a viral vector (data not shown).

### 3.4. New Viruses Are on the Rise

**Figure 2:**
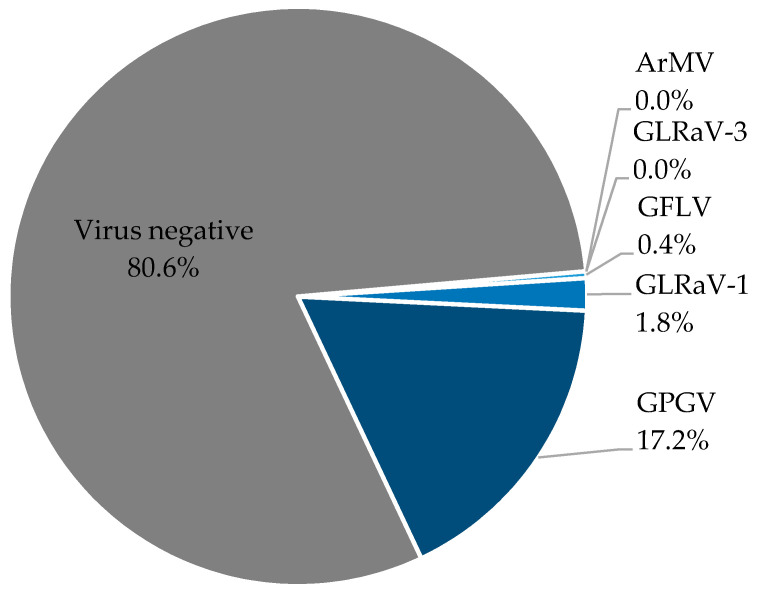
*Grapevine Pinot gris virus* (GPGV) was detected in 17.2% of scion samples from 2018. The graph represents the ELISA results from all scion material tested in 2018. Each sample was tested for the four official viruses ArMV, GFLV, GLRaV-1, and GLRaV-3. Samples were additionally tested for *Grapevine Pinot gris virus* (GPGV) (dark blue). None of the samples was positive for ArMV or GLRaV-3.

In 2018, grapevine samples were also tested for *Grapevine Pinot gris virus* (GPGV), a newly emerged *Trichovirus*, whose symptoms were reported in the sampling area of the WBI [16]. The results presented here point out that GPGV is by far the most abundant virus in scions, with a proportion of 17.2% (Figure 2). In contrast to that, GLRaV-1 was found in only 1.8% of the samples and GFLV in 0.4%. ArMV and GLRaV-3 were not detected in any sample in 2018. A Chi-square test confirmed that these virus frequencies differ significantly, *X*^2^ (4, N = 1337) = 665.52, *p* < 0.01.

### 3.5. Scion Versus Rootstock Material

Rootstock material is also part of the official virus tests. It is analyzed the same way as scion material but is additionally tested for *Grapevine fleck virus* (GFkV). In contrast to scion planting material, rootstock samples show fewer virus infections (Table 3). In total, 4906 pooled rootstock samples were tested between 2009 and 2020. Two of those samples were infected with GFLV and three with ArMV, corresponding to an infection rate of 0.1%. The most abundant virus was GFkV, detected in 0.5% of the samples. Viruses associated with leafroll were not found. GPGV was only tested in 2018 with four positive rootstocks (0.1%, result not shown). Taken together, 99% of rootstock material was virus free compared to 96% of scion material. These frequencies were significantly different, *X*^2^ (1, N = 23662) = 165.23. *p* < 0.001.

## 4. Discussion

This study presents the results of the official virus testing carried out according to the German Wine Growing Ordinance within Germany’s southwestern regions Baden and Wuerttemberg. This official testing allowed the monitoring of virus infestations in vine nurseries of different planting material categories over a time span of twelve years. The results presented here demonstrate that GLRaV-1 was, with few exceptions, the most abundant virus throughout all years and categories, with an occurrence of more than 50% in all virus-positive tested scion samples. Since plant material is screened and selected continuously for the absence of viruses, one should expect the number of virus-positive plants to continuously decrease. In contrast to this, the results presented in this study indicate that virus-positive plants are not reduced over the years but seem to appear in a rather cyclic manner. Since the grapevine cultivar Lemberger is a known carrier of GLRaV-1 and this variety is commonly cultivated, particularly in Wuerttemberg, it might represent a reservoir for this virus [15]. Consequently, the observed pattern could be caused by a sampling bias due to regions inside the probed areas with higher GLRaV-1 incidence. Indeed, scion samples probed in Wuerttemberg (3.8%) showed higher GLRaV-1 incidences than samples from Baden (1.5%). However, GFLV was also found to be more widespread in samples from Wuerttemberg (2.3%) than from Baden (0.9%). This becomes particularly visible in samples from pre-basic and basic material, underlining the higher distribution of both viruses in Wuerttemberg. Therefore, and since the diagnostic laboratory had no influence on sample selection, the patterns seem to be rather random.

Nevertheless, none of the virus rates, except the one of GLRaV-3, decreased to 0%. Interestingly, GLRaV-3 was present in almost none of the samples, whereas GLRaV-1 was the most abundant virus. The leafroll viruses GLRaV-1 and -3 belong to the genus *Ampelovirus* and have multiple vector insects. Three soft scale insects and seven mealybug species are known vectors of GLRaV-1 [17]. Furthermore, eight species are known to transmit GLRaV-3 [18,19]. All vectors of GLRaV-1 were found to transmit GLRaV-3 except *Parthenolecarnium corni* (Bouché) [20]. However, it is rather unlikely that *P. corni* is the only widespread vector of leafroll-associated viruses in the sampling areas of this study. A more likely possibility may be a pool of plants infected exclusively with GLRaV-1, acting as a starting point for the distribution by insects. One source may be vineyards planted with already infected plants surrounding the nurseries. In 2009, such a virus repository was detected when a vineyard of pre-basic propagating material had 64 positive GLRaV-1 samples (Table 1). GLRaV-3 was probably never introduced to the tested regions to the same extent as GLRaV-1. Therefore, its vectors cannot ingest the virus and the infection rates remain low. The modest proportions of GFLV and ArMV may then be explained by the fact that both are assigned to the genus *Nepovirus*, and are transmitted by *Xiphinema index* and *Xiphinema diversicaudatum*, respectively [7]. Nematodes are rather slow virus transmitters due to their slow migration velocity. Additionally, areas must test negative for these nematodes before they are approved for propagation. This measure minimizes the likelihood that viruses will encounter their specific hosts.

Furthermore, what is noticeable about the results of this study is that certified planting material shows by far the highest infection rate of GLRaV-1, with approximately 8% of positive samples. This should not be surprising since those plants are tested only once in ten years. However, it is alarming since only every twentieth plant is sampled in this category and ten plants are pooled into one sample. One can expect that the actual infestation is much higher in those vineyards. Le Maguet et al. [21,22] impressively proved how fast scale-borne viruses could spread inside vineyards. Within seven and four years, infection rates rose from 5% to 85% and from 5% to 50%, respectively. These studies indicate the absolute necessity of virus-free planting material in regions where the corresponding vectors are present. The occurrence of GLRaV-1 vectors in German vineyards is also verified by the results from the retested vine nurseries in this study. Nine percent of those vineyards showed infections in the second test. For seven (6%) vineyards, it was the first documented GLRaV-1 infection, while three (3%) vineyards already had one or two infected samples during the first tests. This indicates a new virus introduction from outside or a missed removal of infected plants acting as virus reservoirs. Although these are not very high percentages, we should move our attention to the future trend, since virus spreading may increase exponentially.

The high proportion of GLRaV-1-infected plants in nurseries for certified material is also alarming since all nurseries are regularly checked visually. Therefore, it can be assumed that many infected grapevines show no symptoms, since otherwise they should have been removed immediately. Furthermore, these assumptions cast doubt on the use of planting material of the standard category whose phytosanitary status is evaluated solely visually [4]. Latently infected plants are a great multiplier for virus infections, considering that approximately twenty cuttings can be harvested from one mother plant.

Pathogens that remain latent in their hosts definitely pose a risk, especially if they are still unknown. One prerequisite for PCR and ELISA testing is to know at least part of the pathogen’s genetic code or protein structures. Undetected pathogens in plant material are distributed unintentionally and their occurrence is artificially upregulated [23]. The longer infected material is distributed unknowingly, the harder it becomes to contain the pathogen. New emerging pathogens can be identified using genetic screening methods, as has been done for *Grapevine Pinot gris virus* (GPGV). Although short RNA readings of symptomatic plants identified the virus, it was rapidly found that GPGV can also be latent in plants [16,24]. The virus test done in 2018 at the WBI proves that GPGV is already highly distributed in German nurseries (17.2%). It was already reported that GPGV is present in Germany, but not the quantity [25]. Additionally, it seems that an increasing number of commercially cultivated grapevines become symptomatic, but this is currently still under investigation at the WBI (Messmer, unpublished data). However, as long as GPGV-infected vines remain latent in nurseries, the virus will continue to spread secretly. A similar incident was recently reported for the *Grapevine red blotch-associated virus* (GRBaV), the causative agent of red blotch disease (RBD) [26,27]. Cases like these indicate the importance of investments in preventive measures to continuously monitor plants’ phytosanitary status with state-of-the-art methods.

Contrary to scion material, rootstock plants show relatively low virus incidences. Only 1% of the analyzed, pooled samples were infected. The nepoviruses GFLV and ArMV, the *Maculavirus* GFkV and also the *Trichovirus* GPGV were detected, but in rather low rates. No ampeloviruses were detected. Since the devastating *Phylloxera* epidemic in the 1860s, American grape varieties have been used almost exclusively as rootstocks because of their tolerance to *Daktulusphaira vitifoliae* (Fitch) [28]. Some of these varieties are known to be tolerant to nematodes, explaining the very low incidence of GFLV and ArMV [29]. However, no tolerances or resistances of American grapevines to scales and mealybugs are known to date. A likely explanation for lower virus infections could be the location of the rootstock vine plots, which are often located away from other vineyards and are more likely to be near farmland. In these areas, vine-specific pests may be less common and vine disease infection pressure is lower. Scion plots, on the other hand, are usually surrounded by commercial vineyards, which allows for a shorter disease transmission range.

In summary, the present study has shown that the monitored viruses are still widely distributed in south-western Germany. Notably, certified planting material had higher GLRaV-1 infestation levels than pre-basic and basic material, suggesting either carryover of unrecognized virus-infected propagation material or viral input from neighboring infected vineyards by its fast moving vectors. However, this result proved the urgency of continuous and close monitoring of the phytosanitary status of planting material. We are aware that within this study only a total of five viruses were investigated, which represent only a small proportion of the viruses from grapevine. In particular, the results from 2018 show that the recently discovered GPGV emerged rather unnoticed to become the most widespread virus in German vineyards, as it is mostly latent in plants. Therefore, it should be considered whether it would not make sense to include viruses like GPGV or GRBaV in the vine planting material regulation, especially since the latter can easily be mistaken for leafroll in optical inspections [23]. In addition, it would be interesting to investigate to what extent GRBaV is already latent in grapevines in Germany. However, global warming is not only shifting climate regions along latitudes, as habitats of insects that are potential pests or vectors of pathogens will also be displaced. The worldwide trade accelerates the possible spread of new dangers, whether pathogens or insects [3,30]. Under these rapidly changing circumstances, it is therefore likely that the monitoring of phytosanitary status will present us with new challenges in the future.

## Figures and Tables

**Table 1:** ELISA results of scion propagation material from 2009–2020, separated by categories. The table shows the amount of positive tested samples for each virus and the percentage relative to the total number of pooled samples of the year in question (square brackets). Samples were tested for *Grapevine fanleaf virus* (GFLV); *Arabis moasic virus* (ArMV); *Grapevine leafroll asscoiated virus 1* (GLRaV-1); *Grapevine leafroll associated virus 3* (GLRaV-3). Listed are the virus tests of pre-basic (a), basic (b), and certified plant material categories (c). Individual years are color coded depending on the virus incidences: 0.0% (green); 0.1–0.9% (pale yellow); 1.0–1.9% (yellow); 2.0–4.9% (dark yellow); 5.0–9.9% (orange); over 10.0% (dark orange).

(**a**)													
**Category**	**Pre-Basic**												**Total**
**Year**	2009	2010	2011	2012	2013	2014	2015	2016	2017	2018	2019	2020	
**No. of pooled samples**	637	733	1059	876	462	499	2927	877	447	457	413	490	9877
**GLRaV-1** **[%]**	65 [10.2]	0 [0.0]	1 [0.1]	1 [0.1]	8 [1.7]	1 [0.2]	9 [0.3]	10 [1.1]	11 [2.5]	8 [1.8]	8 [1.9]	1 [0.2]	123 [1.2]
**GLRaV-3** **[%]**	0 [0.0]	0 [0.0]	1 [0.1]	0 [0.0]	0 [0.0]	0 [0.0]	0 [0.0]	0 [0.0]	0 [0.0]	0 [0.0]	0 [0.0]	0 [0.0]	1 [<0.1]
**GFLV** **[%]**	6 [0.9]	10 [1.4]	1 [0.1]	1 [0.1]	0 [0.0]	1 [0.2]	35 [1.2]	2 [0.2]	1 [0.2]	1 [0.2]	30 [7.3]	1 [0.2]	89 [0.9]
**ArMV** **[%]**	0 [0.0]	14 [1.9]	0 [0.0]	0 [0.0]	0 [0.0]	1 [0.2]	4 [0.1]	12 [1.4]	2 [0.4]	0 [0.0]	0 [0.0]	0 [0.0]	33 [0.3]
(**b**)													
**Category**	**Basic**												**Total**
**Year**	2009	2010	2011	2012	2013	2014	2015	2016	2017	2018	2019	2020	
**No. of pooled samples**	217	251	695	375	301	392	323	848	1042	771	400	1045	6660
**GLRaV-1** **[%]**	0 [0.0]	0 [0.0]	30 [4.3]	5 [1.3]	4 [1.3]	0 [0.0]	0 [0.0]	7 [0.8]	26 [2.5]	16 [2.1]	2 [0.5]	0 [0.0]	90 [1.4]
**GLRaV-3** **[%]**	0 [0.0]	0 [0.0]	0 [0.0]	0 [0.0]	0 [0.0]	0 [0.0]	0 [0.0]	0 [0.0]	0 [0.0]	0 [0.0]	0 [0.0]	0 [0.0]	0 [0.0]
**GFLV** **[%]**	3 [1.4]	12 [4.8]	5 [0.7]	1 [0.3]	5 [1.7]	12 [3.1]	0 [0.0]	9 [1.1]	8 [0.8]	4 [0.5]	11 [2.8]	41 [3.9]	111 [1.7]
**ArMV** **[%]**	1 [0.5]	1 [0.4]	1 [0.1]	0 [0.0]	0 [0.0]	0 [0.0]	0 [0.0]	1 [0.1]	4 [0.4]	0 [0.0]	0 [0.0]	0 [0.0]	8 [0.1]
(**c**)													
**Category**	**Certified**												**Total**
**Year**	2009	2010	2011	2012	2013	2014	2015	2016	2017	2018	2019	2020	
**No. of pooled samples**	2	nt	24	128	1114	242	148	164	139	109	87	62	2219
**GLRaV-1** **[%]**	0 [0.0]	nt	0 [0.0]	13 [10.2]	107 [9.6]	27 [11.2]	8 [5.4]	5 [3.0]	2 [1.4]	0 [0.0]	7 [8.0]	8 [12.9]	177 [8.0]
**GLRaV-3** **[%]**	0 [0.0]	nt	0 [0.0]	0 [0.0]	0 [0.0]	1 [0.4]	0 [0.0]	0 [0.0]	0 [0.0]	0 [0.0]	0 [0.0]	2 [3.2]	3 [0.1]
**GFLV** **[%]**	0 [0.0]	nt	1 [4.2]	4 [3.1]	9 [0.8]	3 [1.2]	8 [5.4]	1 [0.6]	0 [0.0]	0 [0.0]	0 [0.0]	3 [4.8]	29 [1.3]
**ArMV** **[%]**	0 [0.0]	nt	4 [16.7]	0 [0.0]	11 [1.0]	1 [0.4]	0 [0.0]	7 [4.3]	2 [1.4]	0 [0.0]	0 [0.0]	3 [4.8]	28 [1.3]

nt = not tested.

**Table 2:** GFLV and GLRaV-1 occur more frequently in Wuerttemberg than in Baden. Listed are results of ELISA tests on scion propagation material from 2009–2020, separated by the regions Wuerttemberg (a) and Baden (b). The table shows the amount of positive tested samples for each virus and the percentage relative to the total number of pooled samples (square brackets). Individual viruses are color coded depending on their incidences: 0.0% (green); 0.1–0.9% (pale yellow); 1.0–1.9% (yellow); 2.0–4.9% (dark yellow); 5.0–9.9% (orange); over 10.0% (dark orange).

**(a)**						
		**GFLV**	**ArMV**	**GLRaV-1**	**GLRaV-3**	**Total Samples**
Wuerttemberg	Pre-basic [%]	31 [2.7]	1 [0.1]	30 [2.6]	1 [0.1]	**1139**
	Basic [%]	102 [2.6]	4 [0.1]	127 [3.2]	0 [0.0]	**3911**
	Certified [%]	16 [1.1]	16 [1.1]	92 [6.3]	2 [0.1]	**1468**
	**Total [%]**	**149 [2.3]**	**21 [0.3]**	**249 [3.8]**	**3 [<0.1]**	**6518**
**(b)**						
		**GFLV**	**ArMV**	**GLRaV-1**	**GLRaV-3**	**Total Samples**
Baden	Pre-basic [%]	69 [0.8]	22 [0.3]	86 [1.0]	0 [0.0]	**8541**
	Basic [%]	22 [0.9]	3 [0.1]	9 [0.4]	0 [0.0]	**2348**
	Certified [%]	22 [1.2]	13 [0.7]	99 [5.4]	2 [0.1]	**1825**
	**Total [%]**	**113 [0.9]**	**38 [0.3]**	**194 [1.5]**	**2 [<0.1]**	**12,714**

**Table 3:** Rootstock planting material has fewer virus infections than scion material. Listed are the results of ELISA tests on scion and rootstock planting material between 2009 and 2020. The table shows the amount of positive tested samples for each virus and the percentage relative to the total number of pooled samples of the year in question (square brackets). Grapevine fleck virus (GFkV) was only tested in rootstock samples. Individual viruses are color coded depending on their incidences: 0.0% (green); 0.1–0.9% (pale yellow); 1.0–1.9% (yellow); 2.0–4.9% (dark yellow); 5.0–9.9% (orange); over 10.0% (dark orange).

	GLRaV-1	GLRaV-3	GFLV	ArMV	GFkV	No Virus	Total Samples
Scion [%]	390 [2.1]	4 [<0.1]	229 [1.2]	69 [0.4]	nt	18,064 [96.3]	18,756
Rootstock [%]	0 [0.0]	0 [0.0]	2 [0.1]	3 [0.1]	25 [0.5]	4876 [99.4]	4906

nt = not tested.
